# Sleep-Related and Inflammation-Related Indirect Pathways in the Association Between Eczema and Depression: A Cross-Sectional Analysis of the National Health and Nutrition Examination Survey (NHANES) Database

**DOI:** 10.7759/cureus.113087

**Published:** 2026-07-21

**Authors:** Danli Zhong

**Affiliations:** 1 Department of Dermatovenereology, The Seventh Affiliated Hospital, Sun Yat-sen University, Shenzhen, CHN

**Keywords:** atopic dermatitis, depression, nhanes, sleep disorder, systemic inflammation

## Abstract

Background: Eczema/atopic dermatitis (AD) is associated with depression, but the potential indirect pathways involving sleep disorders and peripheral inflammation remain uncertain. We examined sleep-related and inflammation-related indirect effects in the eczema/AD-depression association.

Methods: We analyzed cross-sectional data from 2,071 adults aged 20-59 years who participated in the 2005-2006 National Health and Nutrition Examination Survey. Depression was defined as a Patient Health Questionnaire-9 score ≥10. Physician-diagnosed sleep disorder, the systemic immune-inflammation index (SII), and the aggregate index of systemic inflammation (AISI) were evaluated as potential indirect pathways. Survey-weighted linear probability models and 5,000 design-based Rao-Wu bootstrap replicates were used to estimate indirect effects on the risk-difference (RD) scale. Inflammation-related effects were additionally assessed using equivalence testing.

Results: Eczema/AD was associated with a higher prevalence of depression (total-effect RD = 0.0689, 95% bootstrap confidence interval (CI) 0.0233-0.1142; two-sided P = 0.002). The sleep-related indirect estimate was positive but imprecise (RD = 0.00860, 95% bootstrap CI -0.00024 to 0.01888; two-sided P = 0.058). Indirect effects involving SII or AISI were negligible, and their 90% CIs were contained within the prespecified equivalence bounds of ±0.00689. Similar findings were obtained for the neutrophil-to-lymphocyte ratio, eosinophil count, total immunoglobulin E, and high-sensitivity C-reactive protein.

Conclusions: The findings are consistent with a possible sleep-related indirect pathway between eczema/AD and depression, although the estimate did not meet the conventional two-sided significance threshold. Indirect effects involving the measured peripheral inflammatory markers were negligible relative to the prespecified equivalence margin. Because of the cross-sectional design, temporal ordering and causal mediation cannot be established.

## Introduction

Atopic dermatitis (AD) affects an estimated 204 million people worldwide [1,2] and confers a 1.5- to 2.0-fold increase in the risk of depression [3,4]. Elucidating the mechanisms that bridge AD to depression is essential for developing targeted preventive strategies.

Sleep disturbance is among the most plausible mediators [5]. Hu and colleagues reported that a composite sleep disturbance score mediated 21.2% of the AD-depression association [6]. Whether a clinically diagnosed sleep disorder, reflecting pathology sufficiently severe to prompt medical attention, mediates a comparable proportion remains unknown.

A parallel line of evidence implicates systemic inflammation. Sleep deprivation activates innate immune pathways, elevating circulating cytokines that modulate mood [7,8]. Yin et al. identified a significant mediation pathway from sleep disturbance through inflammatory markers to depressive symptoms in the general population [9]. These observations generate a testable hypothesis: that systemic inflammation may lie downstream of sleep disruption in the AD-depression cascade.

Two critical gaps remain. First, the sleep and inflammatory pathways have never been examined together within a single analytic framework in AD, so their relative contributions and whether they operate as serial or parallel routes are unknown. Second, AD-related inflammation is predominantly Th2-driven, and whether composite blood-count indices such as the systemic immune-inflammation index (SII) and the aggregate index of systemic inflammation (AISI) carry any mediating signal in AD is unclear.

To address these gaps, we specified three pathways before fitting the mediation models: through physician-diagnosed sleep disorder alone, through a serial sequence from sleep disorder to a measured peripheral inflammatory marker, and through the inflammatory marker alone. This ordering was based on the hypothesised clinical sequence; it was not selected by comparing model fit, was not prospectively registered, and cannot establish temporal ordering in cross-sectional data.

## Materials and methods

Study design and population

We analyzed cross-sectional data from the 2005-2006 cycle of the National Health and Nutrition Examination Survey (NHANES). Among 3,409 age-eligible adults aged 20-59 years, 2,071 had complete data on eczema/AD, depression, sleep disorder, SII, AISI, the prespecified covariates, and survey design variables and formed the primary analytic sample.

Exposure and outcome

Eczema/AD was defined by an affirmative response to 'Has a doctor or other health professional ever told you that you have eczema?' (NHANES allergy questionnaire item AGQ180). This item may capture eczematous conditions other than clinically confirmed AD. The direction of resulting bias cannot be assumed: exposure misclassification may affect total and indirect estimates in either direction, particularly when reported physician diagnoses of eczema and sleep disorder share determinants such as healthcare access and diagnostic propensity. We therefore use 'eczema/AD' throughout. Depression was assessed with the Patient Health Questionnaire-9 (PHQ-9) [10]; the primary outcome was PHQ-9 ≥ 10.

Mediators

Sleep disorder was defined by an affirmative response to 'Have you ever been told by a doctor that you have a sleep disorder?' (NHANES sleep disorders questionnaire item SLQ050), capturing clinically recognized sleep pathology rather than subjective sleep quality. SII was calculated as: $\mathrm{SII}=\frac{\mathrm{Neutrophil}\times\mathrm{Platelet}}{\mathrm{Lymphocyte}}$, while AISI was calculated as: $\mathrm{AISI}=\frac{\mathrm{Neutrophil}\times\mathrm{Monocyte}\times\mathrm{Platelet}}{\mathrm{Lymphocyte}}$, and they were selected as pragmatic, reproducible indices of peripheral systemic inflammation because their components were available in NHANES; both were log-transformed. They were not intended as substitutes for AD-specific cytokines, skin biomarkers or measures of neuroinflammation. Neutrophil-to-lymphocyte ratio (NLR), eosinophil count, total immunoglobulin E (IgE), and high-sensitivity C-reactive protein (hs-CRP) were examined in marker-specific sensitivity analyses.

Covariates

All models were adjusted for age group, sex, race/ethnicity, education, poverty status, body mass index category, smoking, alcohol use, physical activity, sleep duration, and history of hypertension, diabetes, asthma, and hay fever. Marital status was not included in the final models.

Statistical analysis

All analyses accounted for the NHANES complex survey design using the survey package version 4.5 (Thomas Lumley, University of Auckland, Auckland, New Zealand) in R version 4.4.2 (R Foundation for Statistical Computing, Vienna, Austria), with 2005-2006 examination weights (WTMEC2YR), primary sampling units (PSUs) (SDMVPSU), and strata (SDMVSTRA). The final design comprised 15 strata and 30 PSUs. The analysis seed was 20260716.

We used linear probability models with an identity link to estimate effects on the RD scale. Three indirect effects were calculated: (1) eczema/AD -> sleep disorder -> depression (a_1_ x b_1_); (2) eczema/AD -> sleep disorder -> inflammation -> depression (a_1_ x d_21 _x b_2_); and (3) eczema/AD -> inflammation -> depression (a_2 _x b_2_). For uncertainty estimation, we generated 5,000 Rao-Wu bootstrap replicate weights that preserved the NHANES strata, PSU structure, and examination weights, and refitted all component models in every replicate [11]. Because these survey replicate weights use a design-specific variance factor, replicate estimates were centred and variance-calibrated to the design-based replicate standard error before calculating percentile CIs. All 5,000 replicates were valid for every reported primary path.

The two-sided empirical bootstrap P value and 95% variance-calibrated percentile CI were treated as the primary inferential results. A directional one-sided P value for the sleep-related indirect effect is reported only as a secondary analysis because the analysis plan was not prospectively registered. The proportion of calibrated bootstrap estimates above zero was also calculated directly from the saved replicate-level results.

Equivalence testing using two one-sided tests (TOSTs) was specified for inflammation-involved pathways in the original analysis but was not preregistered. The smallest effect size of interest was 10% of the revised total RD, corresponding to bounds of -0.00689 and +0.00689. Equivalence was assessed using the 90% bootstrap CI and TOST P value. For sensitivity to unmeasured confounding, an E-value was calculated only for the fully adjusted eczema/AD-to-sleep-disorder risk ratio; it was not interpreted as an E-value for the complete indirect effect [12]. Analytic code and replicate-level outputs are available from the corresponding author on reasonable request. Subgroup analyses were post hoc and exploratory. Stratum-specific estimates were not used to infer effect modification in the absence of formal interaction tests, and no multiplicity adjustment was applied.

A causal interpretation of an indirect effect would require no unmeasured exposure-outcome, exposure-mediator, or mediator-outcome confounding; no exposure-induced mediator-outcome confounder; correct model specification; positivity; consistency; and correct temporal ordering [13]. These assumptions cannot be verified in the present cross-sectional data [14]. The estimates are therefore described as associational indirect effects rather than proof of causal mediation.

## Results

Participant characteristics

**Figure 1:**
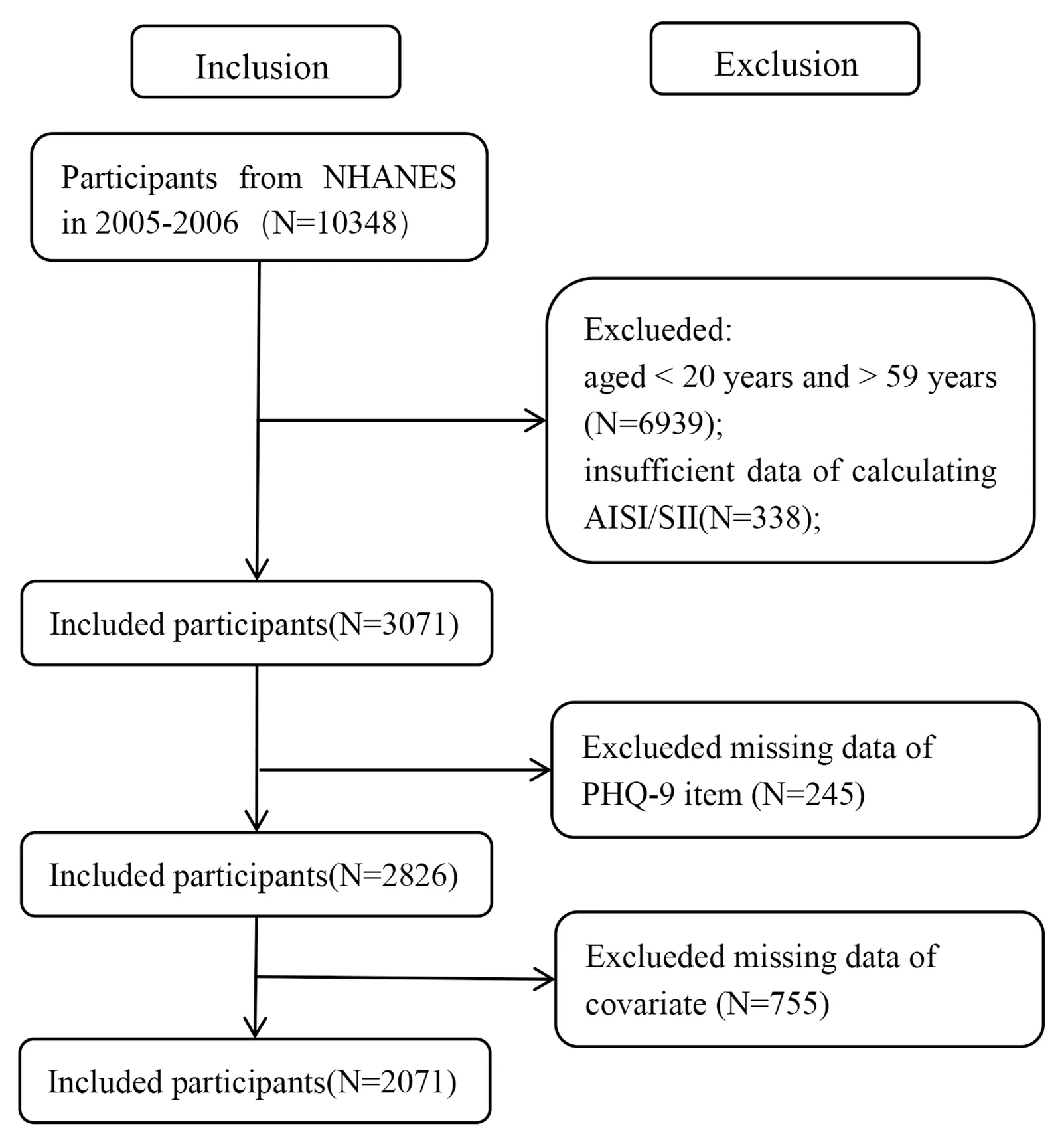
Flow chart of participant selection NHANES: National Health and Nutrition Examination Survey; AISI: aggregate index of systemic inflammation; SII: systemic immune-inflammation index; PHQ-9: Patient Health Questionnaire-9

A total of 2,071 participants formed the complete-case analytic sample (Figure 1). The weighted prevalence of eczema/AD was 8.7%. Participants with eczema/AD had higher prevalences of depression (11.0% vs 4.7%, P < 0.001) and sleep disorder (33.0% vs 21.3%, P = 0.010), with minimal differences in inflammatory indices (Table 1). Compared with the 1,338 age-eligible excluded participants, included participants were younger (weighted mean 38.6 vs 40.3 years), less often male (45.2% vs 56.3%), and less often current smokers (24.8% vs 30.8%); standardized differences suggested potentially meaningful selection on sex and smoking.

**Table 1:** Baseline characteristics of participants with and without AD in the National Health and Nutrition Examination Survey ^1^ Values are presented as unweighted frequencies with weighted percentages for categorical variables and weighted medians (Q1, Q3) for continuous variables. ^2 ^P-values were derived from survey-weighted chi-square tests for categorical variables and survey-weighted rank-sum tests for continuous variables. AD: atopic dermatitis; AISI: aggregate index of systemic inflammation; SII: systemic immune-inflammation index

Characteristic (weighted%)^1^	Overall	Non-AD	AD	Test Statistics	P value^2^
	N = 2,0711	N = 1,9161	N = 1551		
Depression	139 (5.3%)	119 (4.7%)	20 (11%)	F = 16.78	<0.001
SII	540 (405, 756)	539 (403, 749)	569 (414, 825)	F = 2.07	0.058
AISI	289 (195, 425)	286 (195, 421)	295 (201, 449)	F = 1.11	0.3
Sleep disorder	428 (23%)	378 (22%)	50 (33%)	F = 8.78	0.01
Age group				F = 1.81	0.2
20-30	726 (29%)	664 (28%)	62 (35%)	-	-
31-40	537 (27%)	505 (27%)	32 (19%)	-	-
41-59	808 (45%)	747 (45%)	61 (46%)	-	-
Sex				F = 8.32	0.011
Male	894 (45%)	838 (46%)	56 (35%)	-	-
Female	1,177 (55%)	1,078 (54%)	99 (65%)	-	-
Race				F = 6.57	<0.001
Non-Hispanic White individuals	919 (68%)	825 (67%)	94 (79%)	-	-
Non-Hispanic Black individuals	503 (13%)	464 (13%)	39 (10%)	-	-
Mexican American individuals	474 (9.3%)	464 (10.0%)	10 (2.5%)	-	-
Other Hispanic individuals	84 (4.4%)	80 (4.6%)	4 (2.4%)	-	-
Other/Multiracial individuals	91 (5.4%)	83 (5.4%)	8 (5.3%)	-	-
Education				F = 1.60	0.2
< High School	479 (15%)	454 (15%)	25 (11%)	-	-
>= High school	1,592 (85%)	1,462 (85%)	130 (89%)	-	-
Poverty				F = 0.41	0.5
Not poor	1,695 (89%)	1,564 (89%)	131 (90%)	-	-
Poor	376 (11%)	352 (11%)	24 (9.6%)	-	-
BMI group				F = 2.39	0.11
Normal	639 (34%)	578 (34%)	61 (43%)	-	-
Overweight	673 (30%)	635 (31%)	38 (23%)	-	-
Obese	759 (35%)	703 (36%)	56 (34%)	-	-
Smoke				F = 2.17	0.14
Never	1,235 (57%)	1,151 (57%)	84 (51%)	-	-
Former	351 (18%)	323 (18%)	28 (24%)	-	-
Current	485 (25%)	442 (25%)	43 (25%)	-	-
Alcohol				F = 0.71	0.5
Never	766 (31%)	719 (31%)	47 (27%)	-	-
Light	948 (50%)	865 (49%)	83 (56%)	-	-
Moderate/heavy	357 (20%)	332 (20%)	25 (17%)	-	-
How much sleep do you get (hours)?	7.00 (6.00, 8.00)	7.00 (6.00, 8.00)	7.00 (6.00, 8.00)	F = -1.18	0.3
Hypertension	403 (20%)	376 (20%)	27 (20%)	F = 0.09	0.8
Diabetes mellitus	121 (5.2%)	115 (5.3%)	6 (4.4%)	F = 0.20	0.7
Asthma	279 (14%)	248 (13%)	31 (22%)	F = 6.36	0.023
Hay fever	212 (13%)	182 (12%)	30 (22%)	F = 12.22	0.003

Total effect of eczema/AD on depression

Eczema/AD was associated with depression in the fully adjusted survey-weighted logistic model (OR = 3.22, 95% CI 1.91-5.43, P < 0.001). On the RD scale, the total association was 0.0689 (95% variance-calibrated bootstrap CI 0.0233 to 0.1142; two-sided P = 0.002), corresponding to an estimated 6.9-percentage-point higher absolute prevalence of depression.

Mediation through sleep disorder

The sleep-related indirect estimate was positive but imprecise (RD = 0.00860, 95% variance-calibrated bootstrap CI -0.00024 to 0.01888; two-sided P = 0.058). The secondary directional one-sided P value was 0.029, and 97.14% of calibrated bootstrap estimates were above zero. Thus, this estimate did not meet the conventional two-sided significance threshold. The fully adjusted eczema/AD-to-sleep-disorder risk ratio was 1.30 (95% CI 1.02-1.67), corresponding to an E-value of 1.93 for the point estimate and 1.15 for the confidence-limit bound. These E-values apply only to that component association, not to the complete indirect effect.

Non-significant findings for inflammation-involved pathways

Inflammation-involved indirect estimates were small. For SII, the serial estimate was RD = -0.0000076 (95% bootstrap CI -0.0000567 to 0.0000535; two-sided P = 0.608), and the simple SII estimate was RD = -0.0000043 (95% CI -0.001147 to 0.000733; P = 0.787). For AISI, the corresponding serial estimate was RD = 0.0000412 (95% CI -0.0000319 to 0.0001973; P = 0.535), and the simple estimate was RD = 0.000344 (95% CI -0.000514 to 0.001344; P = 0.418). The 90% CIs for all four estimates were contained within the equivalence bounds of +/-0.00689 (all TOST P < 0.001). These results support equivalence only relative to the specified margin and measured markers.

Supplementary analyses

Inflammation-related indirect estimates also remained small for NLR, eosinophil count, total IgE, and hs-CRP, with marker-specific sample sizes of 2,071-2,078; all corresponding 90% CIs fell within the marker-specific equivalence bounds. The earlier multiple-imputation, continuous PHQ-9, and restricted-cubic-spline claims were not retained because they were not required for the reviewer-requested reanalysis and were not supported by a single fully reproducible workflow.

Exploratory subgroup analyses

**Figure 2:**
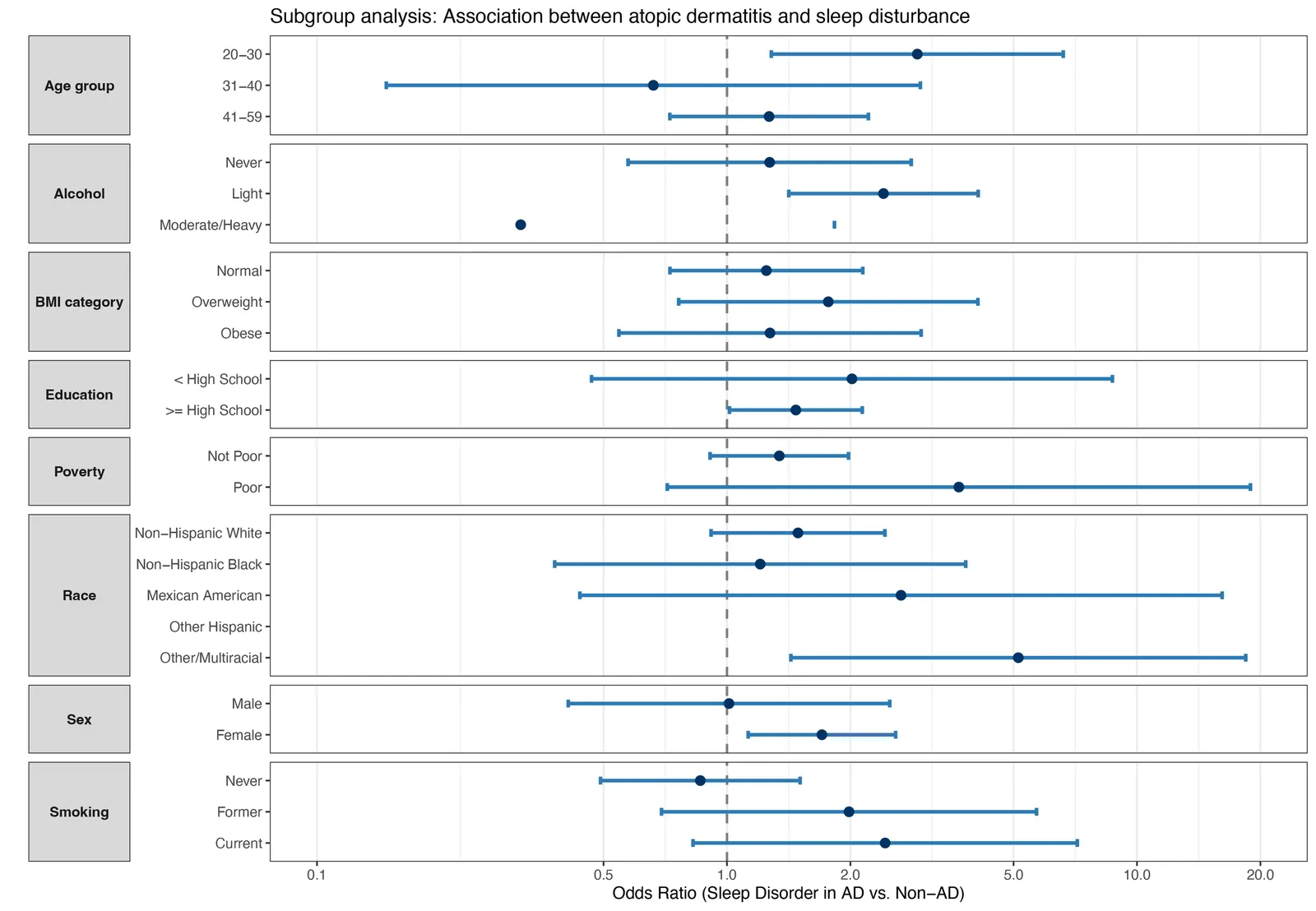
Subgroup analysis of the association between sleep disturbance and atopic dermatitis (AD) across selected demographic and clinical subgroups Forest plot showing the association between eczema/AD and physician-diagnosed sleep disorder across demographic and clinical subgroups, expressed as odds ratios (ORs) with 95% confidence intervals. Each row represents a separate survey-weighted logistic regression model stratified by the subgroup variable and adjusted for all other covariates. The vertical dashed line at OR = 1 indicates the null. The image was created using the forestplot package in R version 4.4.2 (R Foundation for Statistical Computing, Vienna, Austria).

The AD-sleep disorder association was significant among adults aged 20-30 years (OR = 2.91, 95% CI 1.28-6.61), females (OR = 1.70, 95% CI 1.13-2.58), and Other/Multiracial individuals (OR = 5.14, 95% CI 1.43-18.42). These findings should be interpreted cautiously given the small stratum-specific samples (Figure 2).

The subgroup analyses were post hoc and exploratory. Formal design-based Wald interaction tests did not support effect modification by age (P interaction = 0.125), sex (P interaction = 0.251), race/ethnicity (P interaction = 0.915), education (P interaction = 0.471), BMI category (P interaction = 0.804), smoking (P interaction = 0.240), or poverty status (P interaction = 0.119). An interaction with the alcohol category was observed (P interaction = 0.016), but this was one of eight unadjusted exploratory tests and should not be interpreted as confirmatory. The estimate for Other Hispanic participants was not reported because of sparse data and model separation (Figure 2).

## Discussion

Principal findings

In this nationally representative cross-sectional sample of US adults, eczema/AD was associated with a higher prevalence of depression after multivariable adjustment. The estimated indirect effect through physician-diagnosed sleep disorder was positive; however, its 95% bootstrap CI included the null value, and the primary two-sided bootstrap P value did not reach the conventional threshold for statistical significance. Accordingly, the findings are consistent with, but do not establish, a possible sleep-related indirect pathway between eczema/AD and depression.

In contrast, the indirect effects involving the measured peripheral inflammatory markers were very small. For SII and AISI, both the serial pathway through sleep disorder and the simple inflammation-related pathway met the prespecified equivalence criterion relative to the ±0.00689 risk-difference margin. Similar results were observed for NLR, eosinophil count, total IgE, and hs-CRP. These results provide evidence that any indirect effects through these specific measured peripheral markers were negligible relative to the chosen margin; they do not demonstrate that inflammation is unimportant in the broader relationship between eczema/AD and depression.

Comparison with prior work and interpretation

The observed association between eczema/AD and depression is consistent with previous epidemiological evidence. A systematic review and meta-analysis reported that adult AD was associated with higher odds of depression, although definitions of AD and mental-health outcomes varied across studies [15]. Sleep disturbance is common in adults with AD and may arise through nocturnal pruritus, scratching, disease-related discomfort, and psychological distress [16]. A prior small cross-sectional study reported that insomnia symptom severity statistically mediated the association between AD severity and depressive symptoms [17].

The direction of our sleep-related estimate is compatible with this literature, but the smaller and statistically imprecise estimate may reflect important differences in study design and measurement. In particular, our sleep variable captured a physician-recognized sleep disorder rather than insomnia symptoms or sleep quality, and our exposure was based on a self-reported history of physician-diagnosed eczema. These measures may identify a different clinical phenotype from symptom-based assessments of AD severity and insomnia.

The absence of a meaningful indirect effect through the measured peripheral inflammatory indices should also be interpreted cautiously. SII, AISI, and NLR are composite markers derived from circulating blood cell counts, whereas eosinophil count, total IgE, and hs-CRP capture only selected aspects of systemic immune activity. None of these measures directly assesses AD-specific type 2 cytokine activity, lesional skin inflammation, pruritus biology, or neuroinflammatory processes [18,19]. Existing evidence suggests that sleep disturbance, inflammation, and depressive symptoms may be interrelated, but the direction and magnitude of these relationships vary across populations and biomarkers [20].

Thus, our findings should not be interpreted as excluding inflammatory mechanisms. Rather, they suggest that the peripheral markers available in this NHANES cycle did not explain a clinically meaningful proportion of the eczema/AD-depression association under the specified models and equivalence margin. Alternative and potentially overlapping mechanisms may include the itch-scratch-sleep cycle, circadian disruption, psychological stress, social impairment related to visible skin disease, and bidirectional reinforcement between depressive symptoms and sleep disturbance. Reverse causation is also plausible: depression may contribute to sleep problems and may influence eczema symptoms, symptom perception, self-management, or healthcare seeking. Therefore, the hypothesized ordering from eczema/AD to sleep disorder and subsequently to depression should be regarded as a clinically motivated analytic model rather than an established temporal sequence.

Strengths and limitations

This study has several strengths. It used a large, nationally representative US sample and accounted for the NHANES complex survey design. Uncertainty for the indirect effects was estimated with 5,000 Rao-Wu bootstrap replicate weights while refitting all component models in each replicate. We also reported replicate-level directional information, treated two-sided inference as primary, assessed several peripheral inflammatory markers, and used equivalence testing rather than interpreting a non-significant result alone as evidence of no meaningful effect.

Several limitations merit consideration. First, the cross-sectional design precludes determination of temporal ordering and cannot establish causal mediation. A causal interpretation would require strong assumptions regarding confounding, positivity, consistency, model specification, and the absence of exposure-induced mediator-outcome confounding, many of which cannot be verified in these data [13,14]. Second, eczema/AD and sleep disorder were based on self-reported physician diagnoses. The eczema item may include eczematous disorders other than clinically confirmed AD, and the sleep-disorder item does not distinguish between insomnia, obstructive sleep apnea, circadian disorders, or symptom severity. Such misclassification could attenuate AD-specific associations, but the direction of bias is not assured for adjusted total or indirect effects. Because the exposure and sleep mediator both depend on reported clinical diagnosis, healthcare access and diagnostic propensity may also produce differential ascertainment. The estimated total and indirect effects should therefore be interpreted as applying to the NHANES eczema definition rather than confirmed active AD.

Third, NHANES 2005-2006 did not provide detailed information on AD severity, pruritus intensity, disease duration, treatment, anxiety, obstructive sleep apnea, or healthcare utilization. These factors may confound or modify the observed associations. Fourth, the primary analysis used complete cases, and included and excluded participants differed in some characteristics, particularly sex and smoking status; selection bias is therefore possible. Finally, the subgroup analyses were exploratory, involved multiple unadjusted interaction tests, and included sparse data in some strata. The observed alcohol interaction should consequently be interpreted as hypothesis-generating only.

Implications and future directions

The present findings support clinical attention to sleep complaints and depressive symptoms among people with eczema/AD. However, they do not demonstrate that treatment of sleep disorder prevents depression, nor do they establish sleep disorder as a causal mediator. Future longitudinal studies should use clinically confirmed AD, repeated assessments of disease activity, pruritus, sleep characteristics, depressive symptoms, and treatment exposure. Incorporating objective or validated sleep measures and more specific immune biomarkers, including AD-relevant cytokines and skin-based measures where feasible, would help clarify temporal relationships and biological pathways.

## Conclusions

In this cross-sectional NHANES analysis, the sleep-related indirect estimate was positive but did not meet the conventional two-sided significance threshold. Indirect effects involving the measured peripheral inflammatory markers were negligible relative to the prespecified equivalence margin. These findings are consistent with a potential sleep-related pathway but do not establish temporal or causal mediation. Prospective studies with clinically confirmed AD, repeated sleep assessments, and more specific inflammatory biomarkers are needed.
